# Conditional Knockout of *Bmal1* in Corticotropin-Releasing Factor Neurons Does Not Alter Sleep–Wake Rhythm in Mice

**DOI:** 10.3389/fnins.2021.808754

**Published:** 2022-02-18

**Authors:** Chi Jung Hung, Akihiro Yamanaka, Daisuke Ono

**Affiliations:** ^1^Department of Neuroscience II, Research Institute of Environmental Medicine, Nagoya University, Nagoya, Japan; ^2^Department of Neural Regulation, Nagoya University Graduate School of Medicine, Nagoya, Japan

**Keywords:** circadian rhythm, locomotor activity, sleep, *Bmal1*, CRF neurons

## Abstract

Sleep and wakefulness are regulated by both the homeostatic mechanism and circadian clock. In mammals, the central circadian clock, the suprachiasmatic nucleus, in the hypothalamus plays a crucial role in the timing of physiology and behavior. Recently, we found that the circadian regulation of wakefulness was transmitted *via* corticotropin-releasing factor (CRF) neurons in the paraventricular nucleus of the hypothalamus to orexin neurons in the lateral hypothalamus. However, it is still unclear how the molecular clock in the CRF neurons contributes to the regulation of sleep and wakefulness. In the present study, we established CRF neuron-specific *Bmal1*-deficient mice and measured locomotor activity or electroencephalography and electromyography. We found that these mice showed normal circadian locomotor activity rhythms in both light–dark cycle and constant darkness. Furthermore, they showed normal daily patterns of sleep and wakefulness. These results suggest that *Bmal1* in CRF neurons has no effect on either circadian locomotor activity or sleep and wakefulness.

## Introduction

Physiology and behavior, such as sleep and wakefulness, exhibit daily variations that are controlled by the circadian clock. In mammals, almost all cells contain the circadian clock that is thought to generate approximately 24-h rhythms by the transcription–translation feedback loop of several clock genes such as *Bmal1*, *Clock*, *Period*, and *Cryptochrome* (Reppert and Weaver, 2002). The circadian clocks are present in a variety of tissues and cells. Among them, the central circadian clock is located in the suprachiasmatic nucleus (SCN) of the hypothalamus (Moore and Eichler, 1972; Stephan and Zucker, 1972), which has a role for the coordination of the temporal timing of circadian behavioral rhythms (Sawaki et al., 1984; Silver et al., 1996). The SCN comprises thousands of neurons that are neuroanatomically and functionally heterogenous (Van den Pol, 1980; Abrahamson and Moore, 2001). For instance, several neuropeptides, such as arginine vasopressin, vasoactive intestinal peptides, and gastrin- releasing peptides are involved in the SCN. Importantly, almost all SCN neurons are GABAergic, and this inhibitory signal is suggested to be involved in the regulation of circadian outputs such as sleep and wake cycles (Ono et al., 2019, 2020; Maejima et al., 2021). Circadian period, amplitude, and robustness are different in individual SCN neurons without neuronal networks when studied in a dispersed cell culture (Welsh et al., 1995). On the other hand, the circadian rhythms of SCN cells synchronize with each other *via* neuronal networks to make stable and robust circadian rhythms in the SCN (Herzog et al., 2004; Honma et al., 2004).

Previous reports show that the SCN neurons innervate several brain areas (Watts et al., 1987; Watts and Swanson, 1987) and play a role in physiology and behavior (Gizowski et al., 2016; Ono et al., 2020; Paul et al., 2020; Todd et al., 2020; Jones et al., 2021). Importantly, the circadian regulation of wakefulness is transmitted *via* the corticotropin-releasing factor (CRF) neurons in the paraventricular nucleus (PVN) of the hypothalamus to orexin neurons in the lateral hypothalamus (LH) (Ono et al., 2020). Optogenetic activation of CRF neurons in the PVN facilitates wakefulness, and pharmacogenetic suppression and ablation of CRF neurons attenuate wakefulness and locomotor activity, respectively (Ono et al., 2020). These results suggest that circadian information in the SCN transmits through the PVN to the LH pathway.

Although CRF neurons in the PVN are suggested to be involved in circadian-regulated wakefulness, it remains unclear how the molecular circadian clock in the CRF neurons affects sleep and wakefulness. To answer this question, we created CRF neuron-specific *Bmal1*-deficient mice using the Cre-loxp system. We measured locomotor activity, electroencephalogram (EEG), and electromyogram (EMG) to assess circadian rhythms and sleep/wakefulness in CRF neuron-specific *Bmal1*-deficient mice. Circadian locomotor activity rhythms were not different between the CRF neuron-specific *Bmal1*-deficient and control mice. Furthermore, CRF neuron-specific *Bmal1* deficient mice did not show differences in sleep and wakefulness. These results suggest that a deficiency of *Bmal1* in the CRF neurons has no significant effects on circadian locomotor activity and sleep/wakefulness.

## Materials and Methods

### Animals

We used CRF-Cre mice (Itoi et al., 2014), Rosa26-LSL-tdTomato (Ai9) mice (Madisen et al., 2010), and *Bmal1*^flox/flox^ mice (Storch et al., 2007) on C57BL/6J background. All mice were housed in a 12-h dark–light cycle before the experiment (lights on 8:00–20:00). Room temperature (RT) was maintained at 23 ± 2°C, and humidity was maintained between 40 and 70%. Food and water were provided *ad libitum*. All experiments were performed in accordance with the guidelines of the Institutional Animal Care and Use Committees at the Research Institute of Environmental Medicine at Nagoya University (approval numbers: R210729, R210730).

### Locomotor Activity Recording

Both male and female mice were used for locomotor activity recording. The mice were individually housed in a polycarbonate cage placed in a light-tight and air-conditioned box. Spontaneous movements were measured by a passive infrared sensor that detects changes in animal thermal radiation due to movement. The amount of movement was recorded every minute using a computer software (ClockLab, Actimetrics). Free-running period was calculated using *chi*-square periodogram.

### Electroencephalogram and Electromyogram Surgical Procedure

Male mice were anesthetized with 2% isoflurane (095–06573, FUJIFILM Wako Pure Chemical Corporation), and three EEG electrodes (U-1430–01, Wilco) were implanted on the skull, and two EMG electrodes (AS633, Cooner Wire) were attached through the rhomboid muscle (Tsunematsu et al., 2011). Carprofen (Zoetis Inc., Parsippany-Troy Hills, NJ, United States) 20 mg/kg subcutaneous injection was administered the day of and after surgery because of its anti-inflammatory and analgesic properties. After surgery, the mice were housed for about 7 days for recovery. To acclimate the mice to the recording conditions, they were individually connected to a cable with a slip ring (Kissei Comtec Co., Ltd., Matsumoto, Japan) for 7 days before the start of EEG and EMG recordings.

### Sleep/Wake Recordings

Electroencephalogram and EMG signals were filtered at 1.5–30 Hz and 15–300 Hz, respectively, and amplified by an amplifier (AB-610J, Nihon Kohden). Animal behavior was monitored using a charge-coupled device video camera (SPK-E700CHP1, Keiyo Techno) and recorded using an infrared activity sensor (Kissei Comtec). EEG and EMG data were recorded using VitalRecorder with 128 Hz sampling rate (Kissei Comtec) and analyzed with SleepSign software (Kissei Comtec).

### Vigilance State Analysis

Fast Fourier transform (FFT) was performed on the EEG signal; δ (1 ≤ δ < 6 Hz) and θ (6 ≤ θ < 11 Hz) bandwidths were calculated from FFT over a 0–10 Hz window with 1 Hz resolution. Then, EEG and EMG were automatically screened in 4 s epochs by SleepSign and classified as reported in previous studies (Tsunematsu et al., 2011; Hung et al., 2020; Ono et al., 2020). In short, wakefulness was categorized by high EMG amplitude or locomotion score combined with low EEG amplitude; nonrapid eye movement (NREM) sleep was identified by low EMG amplitude with high EEG δ power, and rapid eye movement (REM) sleep was characterized by low EMG and low EEG amplitude with over 50% of θ activity.

### Immunohistochemistry

Mice were anesthetized with isoflurane, and colchicine (3 μl, 10 μg/μl in saline) was injected in the lateral ventricle. About 48 h later, mice were anesthetized and then sequentially perfused with saline and formalin (066–03847, FUJIFILM Wako). Brains were removed and immersed in formalin solution overnight at 4°C, and then immersed in a 30% sucrose solution in phosphate-buffered saline (PBS) for at least 2 days. The brains were frozen in embedding solution (4583, Sakura Finetek Japan) and stored in a −80°C freezer. Brains were subsequently sectioned into 40 μm thick slices using cryostat (CM3050-S, Leica Microsystems K.K.).

Coronal brain sections were washed three times for 10 min in PBS containing 0.25% Triton X-100 (35501–15, Nacalai Tesque) with 1% bovine serum albumin (A7905-500G, MilliporeSigma) (PBS-BX). The brain sections were incubated in PBS-BX with rabbit anti-Bmal1 antibody (1:2,500, NB100-2288; Novus Biologicals, Centennial, CO, United States) and goat anti-CRF antibody (1:200, sc-1761; Santa Cruz Biotechnology, Dallas, TX, United States) overnight at 4°C. Sections were then washed three times for 10 min in PBS-BX and incubated with CF 488-conjugated anti-goat antibody (1:1,000; Biotium Inc., Hayward, CA, United States) and CF 594-conjugated anti-rabbit antibody (Biotium Inc., Hayward, CA, United States) in PBS-BX for 2 h at RT. Sections were then washed in PBS-BX three times for 10 min and stained with 4′,6-diamidino-2-phenylindole (DAPI). After staining, sections were mounted, and the cover-glass was affixed with 50% glycerol in PBS. Photomicrographs were obtained using a microscope (BZ-X700, Keyence) or confocal microscope (LSM710, Carl Zeiss AG).

### Statistical Analyses

Statistical analyses were performed using Statview, Statcel 3, OriginPro 2019 (OriginLab, Northampton, MA, United States) or Excel.

## Results

### Corticotropin-Releasing Factor Expression Patterns in the Brain

**FIGURE 1:**
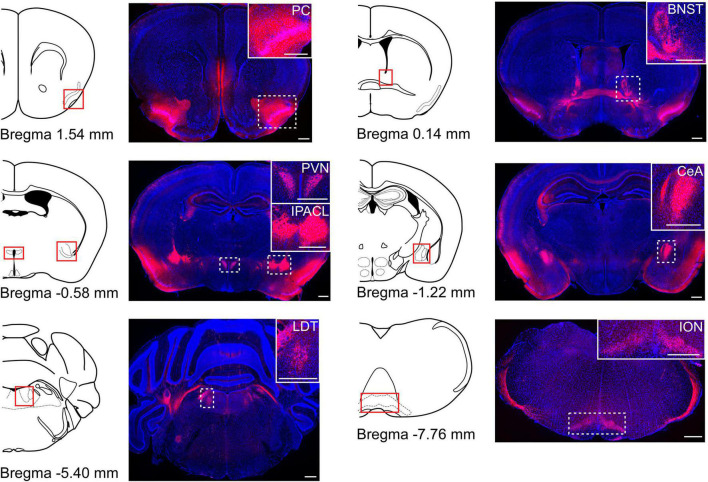
The distribution of CRF neurons in the brain. Cre recombinase specific expression of tdTomato obtained from the CRF-Cre mice crossed with Rosa26-tdTomato reporter mice. Coronal brain sections were stained for DAPI (blue). Schematic drawing of coronal brain slices is shown in the left side of each fluorescence image (modified from Allen brain atlas). Several brain areas expressed tdTomato-positive cells. PC, piriform cortex; BNST, bed nucleus of the stria terminalis; PVN, paraventricular nucleus; IPACL, interstitial nucleus of the posterior limb of the anterior commissure; CeA, central nucleus of the amygdala; LDT, laterodorsal tegmental nucleus; ION, inferior olivary nucleus. White dashed rectangles indicate magnified areas in the panels. Scale bars indicate 500 μm.

To confirm which brain areas expressed CRF in the brain, we crossed CRF-Cre (Itoi et al., 2014) and Rosa26-LSL-tdTomato mice (Madisen et al., 2010). We observed tdTomato-positive cells in several areas in the brain, including the PVN, bed nucleus of the stria terminalis (BNST), central nucleus of the amygdala (CeA), piriform cortex, interstitial nucleus of the posterior limb of the anterior commissure, laterodorsal tegmental nucleus, and inferior olivary nucleus (Figure 1). Some axons of CRF neurons were also observed. These results were consistent with previous reports (Itoi et al., 2014; Walker et al., 2019).

### Generation of Corticotropin-Releasing Factor Neuron-Specific *Bmal1*-Deficient Mice

**FIGURE 2:**
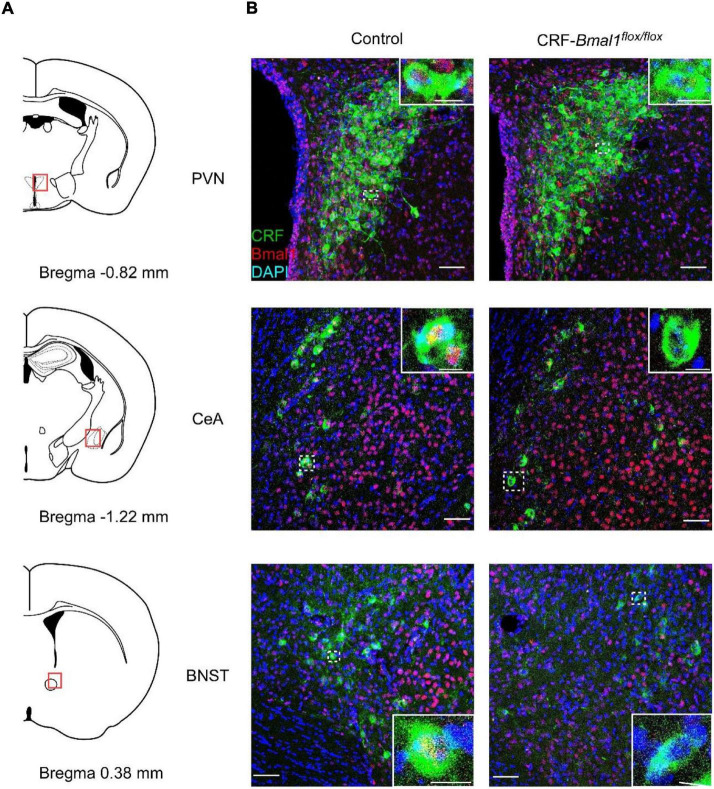
Confirmation of BMAL1 expression in CRF neuron-specific *Bmal1*-deficient mice. **(A)** Schematic drawing of coronal brain slices (modified from Allen brain atlas). **(B)** Fluorescence images of PVN, CeA, and BNST in a *Bmal1*^flox/flox^ (control) or CRF-Cre/*Bmal1*^flox/flox^ mice. Coronal brain sections were immunostained with CRF (green), BMAL1 (red), and DAPI (blue). White dashed rectangles indicate magnified areas in the panels. Scale bars indicate 50 and 10 μm in images with low and high magnification, respectively.

Corticotropin-releasing factor neurons are the key regulators of the hypothalamic–pituitary–adrenal axis, which is involved in the regulation of stress response (Vale et al., 1981). Recently, sleep and wake regulation *via* CRF neurons in the PVN was reported (Li et al., 2020; Ono et al., 2020). Circadian rhythms in the SCN regulate the neuronal activity in the CRF neurons, and the activation of CRF neurons in the PVN further activates the neuronal activity of orexin neurons in the LH (Ono et al., 2020). Although this neuronal circuit is suggested to be involved in the regulation of sleep and wakefulness, it remains unclear whether the circadian clock in the CRF neurons contributes to the regulation of sleep and wakefulness. To answer this question, we crossed CRF-Cre (Itoi et al., 2014) and *Bmal1* flox mice (Storch et al., 2007) to generate CRF neuron-specific *Bmal1* knockout mice (CRF-*Bmal1*^flox/flox^ mice). We obtained CRF-*Bmal1*^flox/flox^ and *Bmal1*^flox/flox^ (control) littermate mice and stained for CRF peptides and *Bmal1* proteins in the brain using immunohistochemistry. Almost all CRF neurons expressed BMAL1 in the control mice (97.5, 97.3, and 91.7% in the PVN, CeA, and BNST, respectively; *n* = 2 mice), whereas *Bmal1* proteins were rarely observed in the CRF peptide-positive neurons in the CRF-*Bmal1^flox/flox^* mice (12.8, 27.8, and 21.2% in the PVN, CeA, and BNST, respectively; *n* = 2 mice) (Figure 2). The number of BMAL1- positive neurons among non-CRF neurons in the PVN was not different between two genotypes (control mice: 91.4%, CRF-*Bmal1^flox/flox^* mice: 90.9%, *n* = 2 mice). We also counted the number of CRF-positive neurons in the BNST, CeA, and PVN of control (BNST: 490 and 505 cells; CeA: 229 and 480 cells; PVN: 338 and 218 cells, *n* = 2 mice) and CRF-*Bmal1*^flox/flox^ (BNST: 242 and 265 cells; CeA: 194 and 138 cells; PVN: 303 and 387 cells, *n* = 2 mice) mice. These results indicate that *Bmal1* expression was deleted in the majority of CRF neurons in the CRF-*Bmal1*^flox/flox^ mice.

### Locomotor Activity Rhythm Was Not Affected by *Bmal1* Deficiency in the Corticotropin-Releasing Factor Neurons

**FIGURE 3:**
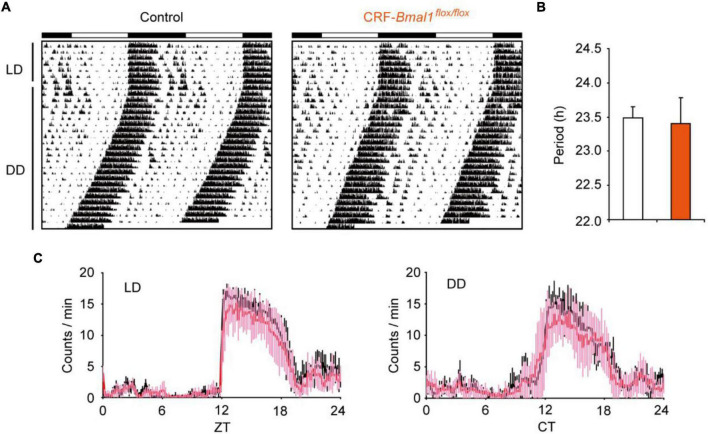
Circadian behavioral rhythms in CRF neuron-specific *Bmal1*- deficient mice. **(A)** Representative example of locomotor activity rhythms in control (male, 2; female, 4) or CRF-Cre/*Bmal1^flox/flox^* (male, 6; female, 1) mice under LD and DD. **(B)** Free-running period calculated by *chi*-square periodogram under DD. **(C)** Daily patterns’ locomotor activity in both genotypes (black: control mice, Pink: CRF-Cre/*Bmal1^flox/flox^* mice). Data are expressed as mean ± SD.

First, we measured locomotor activity using an infrared sensor from control and CRF-*Bmal1*^flox/flox^ mice under light–dark (LD) cycle and constant darkness (DD). The daily patterns of circadian locomotor activity rhythms were not different between control and CRF-*Bmal1*^flox/flox^ mice in both LD and DD conditions (Figures 3A,C). Moreover, the amount of locomotion was not different. The free-running period under DD calculated by *chi*-square periodogram was not different between the two groups (Figure 3B). These results indicate that *Bmal1* deficiency in CRF neurons did not change circadian behavioral rhythms.

### *Bmal1* Deficiency in Corticotropin-Releasing Factor Neurons Did Not Have Significant Effects in Sleep and Wakefulness

**FIGURE 4:**
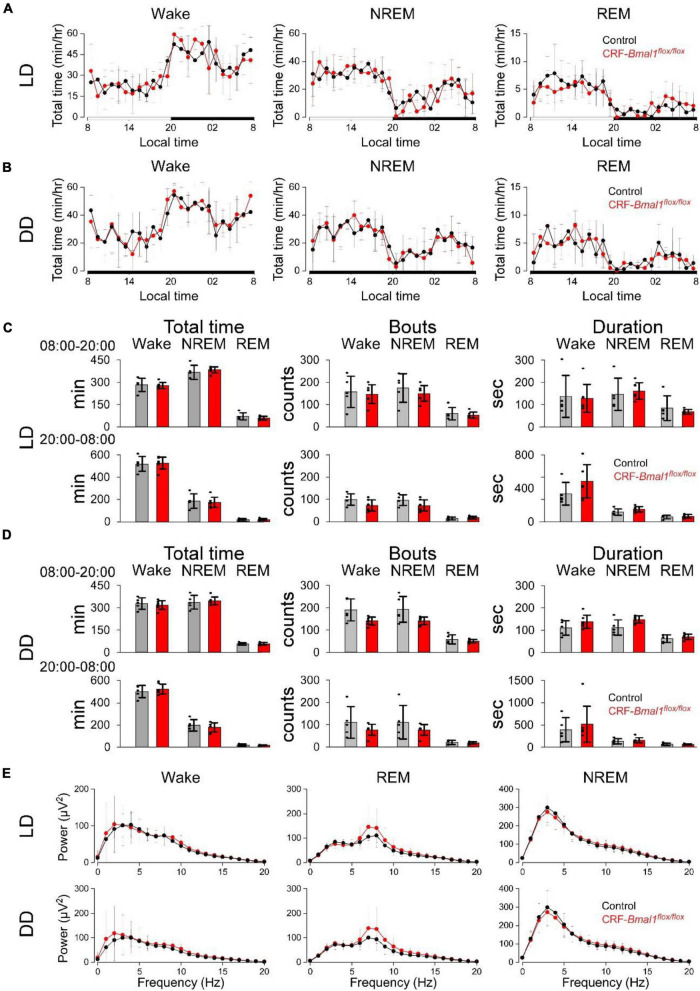
Sleep and wakefulness in CRF neuron-specific *Bmal1* deficient mice. **(A,B)** Daily patterns of wake, NREM, and REM sleep in control (black) (*n* = 7) or CRF-Cre/*Bmal1^flox/flox^* (*n* = 5) mice (red) under LD **(A)** and DD **(B)**. **(C,D)** Total time (left), number of bouts (middle), and duration (right) of wake, NREM, and REM sleep under LD **(C)** and DD **(D)**. **(E)** EEG power during wake, NREM, and REM sleep in both genotypes under LD (top) or DD (bottom). Data are expressed as mean ± SD.

Next, we measured the sleep and wakefulness from control and CRF-*Bmal1*^flox/flox^ mice using EEG and EMG recordings under LD and DD. As for the sleep/wakefulness in DD, we analyzed the EEG and EMG data during the second day of DD. Clear daily patterns of wakefulness, NREM, and REM sleep were observed in both groups under LD and DD (Figures 4A,B). There was no significant difference between the two groups in these daily patterns in LD and DD (Figures 4A,B). The total time, number of bouts, and mean duration of bouts were also not different between the two groups (Figures 4C,D). When we compared the EEG power spectrum, no difference was detected (Figure 4E). These results indicate that *Bmal1* in CRF neurons have essentially no effect on sleep and wakefulness.

## Discussion

We found that *Bmal1* in the CRF neurons was not involved in the regulation of circadian locomotor activity rhythms and was not related to the circadian clock-regulated wakefulness in mice. Importantly, the mice with the whole-body deletion of *Bmal1* exhibit arrhythmic circadian behavior (Bunger et al., 2000) and increases of NREM and REM sleep (Laposky et al., 2005). These results and our findings indicate that *Bmal1* plays a role for the regulation of sleep and wakefulness, but these effects were not related to the CRF neurons in mice.

The CRF neurons in the PVN were previously reported to be important in circadian clock-related wakefulness (Ono et al., 2020). However, the CRF neurons in other regions could also have a role in the modulation of sleep and wakefulness. CRF neurons are observed in several brain areas. Among them, CeA contains several important neurons that regulate sleep (Sanford et al., 2002; Ma et al., 2019) and has several functions such as the control of fear, anxiety, and feeding (Ciocchi et al., 2010; Cai et al., 2014; Fadok et al., 2017). The CRF neurons in the CeA might have a functional role in sleep and wake regulation.

Although the brain contains many neurons that are crucial for the regulation of sleep and wakefulness, peripheral tissues also have a potential for the regulation of sleep. For instance, the restoration of *Bmal1* specifically into the brain in whole-body *Bmal1*-deficient mice did not restore sleep abnormality (Ehlen et al., 2017), whereas the restoration of *Bmal1* in skeletal muscle completely restored NREM sleep duration to wild-type levels, indicating that *Bmal1* in skeletal muscle plays a role in the regulation of sleep and wakefulness. Recently, Jones reported that CRF neuron-specific *Bmal1*-deficient mice dampened corticosterone rhythms (Jones et al., 2021). Since corticosterone administration or the inhibition of corticosterone synthesis modulates sleep and wakefulness (Vazquez-Palacios et al., 2001; Drouet et al., 2011), sleep and wakefulness in mice might also be affected by corticosterone rhythms.

The functional roles of *Bmal1* in sleep and wakefulness have been reported, but other clock genes also play a role in the regulation of sleep. For example, *Dec2* is one of the clock genes in mammals (Honma et al., 2002) that is a negative component of the molecular circadian clock. In humans, familial advanced sleep-phase syndrome is a disorder in which the timing of sleep and the peak period of alertness are advanced several hours relative to the social clock (He et al., 2009). This phenotype is due to point mutation in *Dec2*. Furthermore, this point mutation suppresses prepro-orexin promoter activity (Hirano et al., 2018). Therefore, clock genes in some neurons that are involved in the regulation of sleep and wakefulness have the potential to modulate sleep and wake regulation.

## Conclusion

Corticotropin-releasing factor neurons in the PVN are critical for the regulation of circadian clock-derived wakefulness, but *Bmal1* in the CRF neurons is not essential for the regulation of sleep and wakefulness in mice.

## Data Availability Statement

The raw data supporting the conclusions of this article will be made available by the authors, without undue reservation.

## Ethics Statement

The animal study was reviewed and approved by the Institutional Animal Care and Use Committees at the Research Institute of Environmental Medicine at Nagoya University (approval numbers: R210729 and R210730).

## Author Contributions

DO designed the research. AY contributed in the discussion of the research. DO and CH performed the research, analyzed data, and wrote the manuscript. All authors contributed to the article and approved the submitted version.

## Conflict of Interest

The authors declare that the research was conducted in the absence of any commercial or financial relationships that could be construed as a potential conflict of interest.

## Publisher’s Note

All claims expressed in this article are solely those of the authors and do not necessarily represent those of their affiliated organizations, or those of the publisher, the editors and the reviewers. Any product that may be evaluated in this article, or claim that may be made by its manufacturer, is not guaranteed or endorsed by the publisher.
